# Sex differences in CKD risk factors across ethnic groups

**DOI:** 10.1093/ndt/gfae038

**Published:** 2024-02-08

**Authors:** Taryn G Vosters, Frouke M Kingma, Vianda S Stel, Bert-Jan H van den Born, Brechje J M V Huisman, Frans J van Ittersum, Kitty J Jager, Liffert Vogt, Irene G M van Valkengoed

**Affiliations:** Amsterdam UMC, University of Amsterdam, Department of Public and Occupational Health, Amsterdam Public Health research institute, Amsterdam, The Netherlands; Amsterdam UMC, University of Amsterdam, Department of Public and Occupational Health, Amsterdam Public Health research institute, Amsterdam, The Netherlands; Amsterdam UMC, University of Amsterdam, Department of Medical Informatics, Amsterdam Public Health research institute, Amsterdam, The Netherlands; Amsterdam UMC, University of Amsterdam, Department of Public and Occupational Health, Amsterdam Public Health research institute, Amsterdam, The Netherlands; Amsterdam UMC, University of Amsterdam, Department of Internal & Vascular Medicine, Amsterdam Public Health research institute, Amsterdam, The Netherlands; Amsterdam UMC, University of Amsterdam, Dept Internal Medicine, Section Nephrology, Amsterdam Cardiovascular Sciences, Amsterdam, The Netherlands; Amsterdam UMC, Vrije Universiteit Amsterdam, Department of Nephrology, Amsterdam Cardiovascular Sciences, Amsterdam, The Netherlands; Amsterdam UMC, University of Amsterdam, Department of Medical Informatics, Amsterdam Public Health research institute, Amsterdam, The Netherlands; Amsterdam UMC, University of Amsterdam, Dept Internal Medicine, Section Nephrology, Amsterdam Cardiovascular Sciences, Amsterdam, The Netherlands; Amsterdam UMC, University of Amsterdam, Department of Public and Occupational Health, Amsterdam Public Health research institute, Amsterdam, The Netherlands

To the Editor,

Chronic kidney disease (CKD) poses a substantial public health burden, affecting an estimated 800 million individuals worldwide [1]. Globally, estimated CKD prevalence is higher in women than in men, 14.6% versus 12.8% [2], but this sex disparity may vary between women and men across ethnic groups [3]. Ethnic minority women and men have been reported to suffer from higher burdens of CKD globally than their native counterparts or than the majority population in the countries in which they reside [4].

Prevention of CKD is often aimed at control of risk factors such as hypertension, diabetes mellitus, obesity and smoking. Whether this focus benefits both women and men equally, in general and across different ethnic groups, remains to be determined. The potential impact of current primary prevention methods focusing on these factors may vary. Previous studies have reported sex and ethnic differences in CKD prevalence as well as the association of hypertension, diabetes mellitus, obesity and smoking with unfavorable renal outcome measures [5, 6]. Insight into the contribution of these risk factors to the burden of prevalent CKD across groups may help to determine in which groups current prevention measures should be intensified or possibly adjusted.

We used data from the prospective population-based multi-ethnic Healthy Life in Urban Setting (HELIUS) cohort study [4]. Using the baseline data, we conducted a cross sectional analysis to determine the contribution of hypertension, diabetes, obesity and smoking, defined as cardiovascular risk factors by the European Society of Cardiology and Dutch Cardiovascular Risk management guidelines, to prevalent CKD in women and men across six ethnic groups, including Dutch, South Asian Surinamese, African Surinamese, Ghanaian, Turkish and Moroccan descent populations. Ethnicity was determined by the registered country of birth of the participant, and that of his/her parents. Data was collected between 2011 and 2015 via questionnaire and physical examination from participants aged 18–70 years living in Amsterdam, the Netherlands. CKD was determined by an estimated glomerular filtration rate (eGFR) <60 mL/min/1.73 m^2^ and/or albumin–creatinine ratio ≥3 mg/mmol [7] based on a single sample. The contribution of risk factors was estimated via an adjusted Population Attributable Fraction (PAF) equation {PAF = P[(PR–1)/PR] × 100} proposed by Rockhill *et al*. [8], where P reflects the proportion of those exposed to the risk factor with CKD, while including age-adjusted Poisson regression analyses to calculate the necessary prevalence ratios (PR) of those with CKD in the exposed to the non/exposed groups, needed for the PAF. Finally, we multiplied the PAF for each risk factor separately with the age-adjusted CKD prevalence, to provide an estimate of the risk factor related CKD prevalence for women and men in the total study population and per ethnic group.

The study population consisted of 42.2% men and 57.8% women with a mean age of 45 years and 44 years, respectively (Table 1). Men had higher rates of hypertension and diabetes mellitus overall and across ethnic groups than women. Obesity rates were higher in women than they were in men overall and across ethnic groups. The overall age-adjusted CKD prevalence was higher in women than in men and ranged between 3.3% and 9.5% across groups. These sex differences were bigger in the Ghanaian, Turkish and Moroccan groups than in the Dutch reference group (*P*-value for interaction <.001). In line with this, the age-adjusted prevalence of albuminuria was higher in women than in men in all groups with the exception of the Dutch and South Asian Surinamese groups (Table 1); however, the age-adjusted eGFR <60 mL/min/1.73 m^2^ prevalence was similar in men and women overall, but patterns varied across ethnic groups. The estimated contribution of hypertension and diabetes mellitus to CKD prevalence was higher in men than in women, and this was observed across ethnic groups (range in men 10.0%–65.0%, and in women 2.3%–45.1%; Fig. 1A and B). Overall, women had higher estimated contributions of obesity to CKD than men, and this was also evident in ethnic groups, aside from the Dutch group (range in men 9.1%–22.9%, and in women 7.0%–27.8%; Fig. 1C). No PAF was calculated for smoking, since the PR derived from the Poisson regression analyses was smaller than 1. Hypertension was the strongest contributor to CKD across ethnic groups with a 55.3% and 33.8% estimated contribution overall in men and women, respectively (Fig. 1A). On the other hand, sex differences in the contributions of risk factors to prevalent albuminuria across groups showed similar patterns compared with CKD prevalence estimates—for an isolated eGFR <60 mL/min/1.73 m^2^ the pattern of sex differences in contributions varied across ethnic groups (Supplementary data, Table S1). Sensitivity analyses included a combined estimation of the selected risk factors, which showed higher contributions in men than in women overall and across ethnic groups (Supplementary data, Table S2). Use of the CKD Epidemiology Collaboration 2021 equation, omitting the correction factor for ethnicity, in the Poisson regression as a sensitivity analysis indicated that PAF estimates are not likely to differ from the main results, as the estimated association between risk factors and CKD was similar (Supplementary data, Table S3).

**Figure 1:**
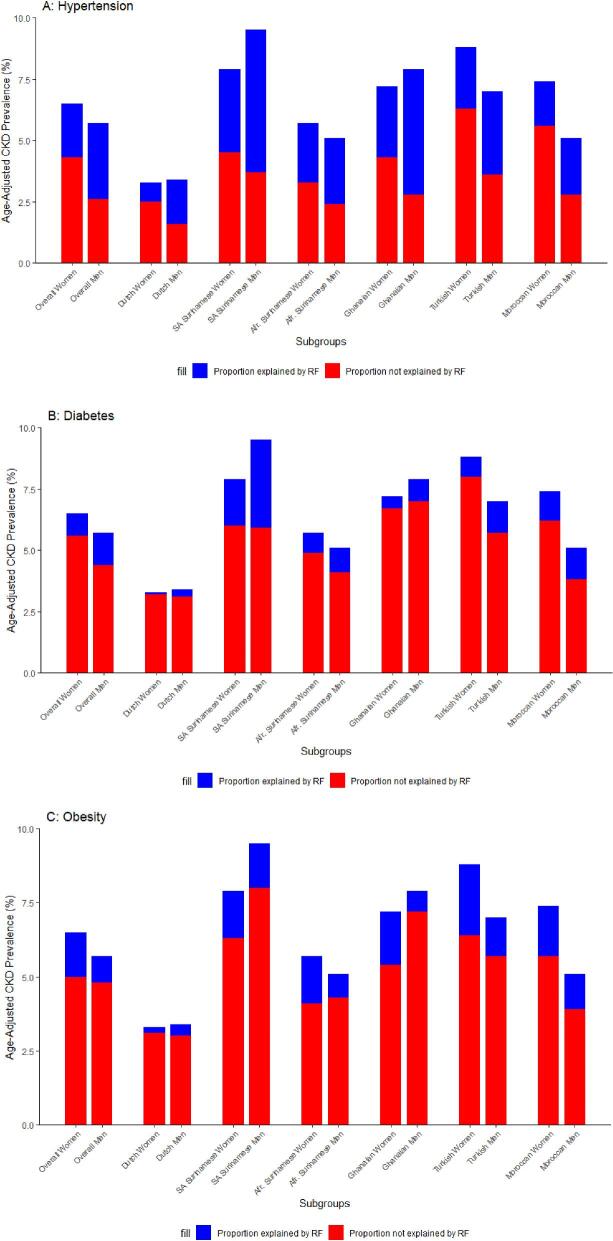
Age-adjusted risk factor contribution to prevalent CKD in women and men across ethnic groups. Population attributable fraction in % per subgroup depicted. CKD prevalence in percentages (*y*-axis), across ethnic groups by sex (*x*-axis), adjusted for age, split between risk factor related (blue) and risk factor unrelated (red) CKD prevalence. Blue and red summed depict the age adjusted CKD prevalence per group. No PAFs were calculated for smoking status due to negative prevalence ratio estimates.

**Table 1:** Baseline characteristics of the study population across ethnic groups, by sex.

	Overall Ppopulation		Dutch (*n* = 4525)		South Asian Surinamese (*n* = 3026)		African Surinamese (*n* = 4105)		Ghanaian (*n* = 2313)		Turkish (*n* = 3578)		Moroccan (*n* = 3883)	
	Men (*n* = 9046)	Women (*n* = 12 384)	Men (*n* = 2069)	Women (*n* = 2456)	Men (*n* = 1363)	Women (*n* = 1663)	Men (*n* = 1599)	Women (*n* = 2506)	Men (*n* = 896)	Women (*n* = 1417)	Men (*n* = 1617)	Women (*n* = 1961)	Men (*n* = 1502)	Women (*n* = 2381)
Age (years)	45 (32–58)	44 (31–57)	48 (35–59)	47 (32–57)	46 (33–56)	48 (37–56)	51 (40–58)	50 (40–57)	49 (41–55)	45 (37–51)	43 (31–50)	41 (30–49)	42 (32–52)	39 (28–50)
Smoking status														
Yes	2886 (32.1)	2227 (18.1)	542 (26.2)	570 (23.3)	540 (39.7)	314 (18.9)	683 (43.0)	610 (24.4)	68 (7.6)	36 (2.6)	661 (41.2)	570 (29.2)	392 (26.3)	127 (5.3)
No, never	3796 (42.2)	8163 (66.2)	709 (34.3)	965 (39.4)	592 (43.6)	1157 (69.8)	560 (35.2)	1433 (57.4)	706 (79.2)	1298 (92.3)	536 (33.4)	1144 (58.7)	694 (46.5)	2168 (91.3)
Former smoker	2321 (25.8)	1939 (15.7)	816 (39.5)	914 (37.3)	227 (16.7)	186 (11.2)	346 (21.8)	452 (18.1)	117 (13.1)	72 (5.1)	408 (25.4)	235 (12.1)	407 (27.3)	80 (3.4)
SBP (mmHg)	131.0 (115.0–147.0)	124.0 (106.0–140.0)	127.0 (119.0–137.5)	118.0 (109.0–128.0)	128.0 (119.5–139.5)	123.0 (112.0–137.5)	131.5 (122.5–143.0)	128.5 (116.0–141.0)	136.5 (127.0–148.5)	131.0 (120.0–144.0)	125.5 (118.0–134.5)	117.0 (108.0–128.2)	125.5 (117.5–135.0)	115.5 (107.5–127.0)
DBP (mmHg)	82.0 (72.0–92.0)	76.0 (65.5–86.5)	80.0 (74.0–87.0)	73.0 (67.5–79.0)	82.0 (75.5–89.0)	77.0 (70.0–84.3)	83.5 (76.5–91.5)	79.5 (72.5–87.5)	86.5 (79.5–94.0)	82.0 (74.5–89.5)	80.5 (74.5–87.0)	74.0 (68.0–80.5)	78.5 (73.0–84.5)	71.5 (66.0–77.5)
Hypertension, *n* (%)	3314 (36.7)	3662 (29.6)	669 (32.4)	457 (18.6)	560 (41.1)	593 (35.7)	743 (46.6)	1135 (45.4)	520 (58.0)	698 (49.3)	480 (29.7)	411 (21.0)	342 (22.8)	370 (15.6)
Fasting glucose (mmol/L)	5.6 (4.3–6.9)	5.3 (4.1–6.5)	5.4 (5.1–5.7)	5.0 (4.8–5.4)	5.5 (5.2–6.2)	5.3 (4.9–5.8)	5.3 (5.0–5.8)	5.1 (4.8–5.6)	5.3 (5.0–5.8)	5.0 (4.7–5.5)	5.4 (5.1–5.8)	5.1 (4.8–5.4)	5.4 (5.1–5.9)	5.1 (4.8–5.5)
Diabetes, *n* (%)	1087 (12.0)	1237 (10.0)	95 (4.6)	50 (2.0)	269 (19.7)	244 (14.7)	168 (10.5)	276 (11.0)	96 (10.7)	113 (8.0)	148 (9.1)	138 (7.0)	161 (10.7)	217 (9.1)
Hypercholesterolemia, *n* (%)	1964 (21.7)	2301 (18.6)	490 (23.7)	532 (21.7)	463 (34.0)	468 (28.1)	303 (18.9)	490 (19.6)	190 (21.3)	222 (15.7)	315 (19.5)	318 (16.2)	195 (13.0)	256 (10.8)
BMI (kg/m^2^)	26.4 (22.2–30.6)	27.7 (21.8–33.6)	25.2 (21.4–29.0)	24.4 (19.9–28.9)	25.8 (21.6–30.0)	26.7 (21.4–32.0)	26.3 (21.9–30.7)	28.8 (22.9–34.7)	26.8 (23.1–30.5	29.6 (24.3–34.9)	27.9 (23.5–32.3)	29.1 (22.6–35.6)	26.7 (22.7–30.7)	28.1 (22.4–33.8)
Obesity, *n* (%)	1570 (17.3)	3848 (31.1)	208 (10.1)	250 (10.2)	186 (13.6)	390 (23.4)	275 (17.2)	941 (37.5)	158 (17.6)	628 (44.3)	454 (28.1)	801 (40.8)	289 (19.2)	838 (35.2)
eGFR (mL/min/1.73 m^2^)	100.0 (84.0–116.0)	104.1 (86.2–122)	97.0 (86.2–107.0)	94.4 (82.4–104.4)	95.7 (84.2–106.2)	100.1 (88.9–109.6)	100.9 (88.2–113.9)	104.6 (91.2–117.4)	98.9 (86.8–111.3)	108.8 (94.2–121.0)	105.4 (96.4–114.3)	109.9 (101.7–119.3)	105.6 (97.1–114.8)	113.4 (104.0–122.6)
eGFR <60 mL/min/1.73 m^2^ (%)	1.7** (1.4–2.1)	1.3 (1.1–1.60	1.4 (1.0–2.4)	1.1 (0.7–1.7)	2.9 (2.0–4.1)	2.4 (1.6–3.4)	1.6 (1.0–2.6)	1.1 (0.7–1.8)	2.8 (1.1–6.4)	1.2 (0.5–7.9)	2.2 (0.9–4.6)	1.2 (0.5–2.8)	0.9 (0.4–1.9)	0.9 (0.4–1.8)
ACR (mg/mmol)	1.25 (1.16–1.34)	1.38 (1.30–1.46)	0.22 (0.14–0.37)	0.27 (0.17–0.42)	0.25 (0.14–0.55)	0.31 (0.19–0.63)	0.22 (0.13–0.45)	0.29 (0.18–0.57)	0.20 (0.12–0.42)	0.28 (0.18–0.61)	0.25 (0.15–0.46)	0.37 (0.22–0.75)	0.26 (0.16–0.49)	0.40 (0.23–0.81)
Albuminuria (%)	4.6 (4.2–5.1)	5.6 (5.2–6.1)	2.3 (1.7–3.4)	2.2 (1.6–3.0)	8.0 (6.5–9.8)	6.3 (5.2–7.8)	4.4 (3.4–5.7)	5.0 (4.1–6.2)	5.5 (3.3–9.1)	6.6 (5.1–12.9)	5.4 (3.9–7.7)	8.3 (6.6–10.6)	5.0 (3.7–6.7)	7.0 (5.7–8.5)
CKD prevalence (%)	5.7* (5.2–6.3)	6.5 (6.0–7.0)	3.4 (2.7–4.6)	3.3 (2.6–4.2)	9.5 (7.9–11.4)	7.9 (6.6–9.6)	5.1 (4.1–6.6)	5.7 (4.7–6.9)	7.9*** (5.0–12.4)	7.2 (5.6–13.6)	7.0*** (5.0–9.9)	8.8 (7.0–11.3)	5.1*** (3.8–6.8)	7.4 (6.1–9.0)

Data are reported as mean (SD), median (25th–75th percentile) or *n* (%).

Obesity: BMI >30 kg/m^2^; CKD prevalence: eGFR <60 mL/min/1.73 m^2^ and/or albumin-to-creatinine ratio (ACR) ≥3 mg/mmol; albuminuria: ACR ≥3 mg/mmol.

*P-value 0.002 for sex difference in overall CKD stage 1–5 prevalence; **P-value 0.139 for the sex difference in overall eGFR <60 mL/min/1.73 m^2^; ***sex difference significantly different from the Dutch.

SBP: systolic blood pressure; DBP: diastolic blood pressure; eGFR: estimated glomerular filtration rate estimated by CKD-EPI_creatinine_; ACR: albumin-to-creatinine ratio in urine; BMI: body mass index.

In conclusion, the contribution of hypertension and diabetes mellitus to the observed CKD prevalence was higher in men than women across ethnic groups, with hypertension contributing the most. The opposite pattern was observed for obesity, as higher estimated contributions were seen in women than in men. The high contribution of hypertension, and the higher contribution in men than women for diabetes and hypertension, is in line with some studies [9, 10], but not others [1, 11]. Previous work has investigated the contribution of obesity, however no studies have assessed whether the contribution differs between men and women. The sex differences might be explained by differences in the pathophysiology [12] treatment, health behaviors [13] or underestimation due to a lack of sex-specific cut-off values [14]. The lack of contribution of smoking might in part be explained by reporting bias, selective non-response or competing risk of death in smokers [15].

Importantly, while findings confirm the benefit of current prevention strategies, e.g. treatment of high blood pressure [7], we show that these will not fully eliminate sex differences in prevalent CKD. We acknowledge that this presumes causality, complete confounding adjustment and a perfect intervention [8]. Additionally, baseline measurements were based on single measurements, although clinical practice recommends a repeat measurement to confirm chronicity of disease. Furthermore, we recognize that hypertension may be a cause as well as an effect of CKD. Nevertheless, we consider that in specific ethnic subgroups of women and men, additional factors may play a role, such as more frequent hypertensive complications during pregnancy, early menopause, dietary factors including potentially nephrotoxic herbal supplementation, environmental factors, social stressors or occupational exposures [7].

## Supplementary Material

gfae038_Supplemental_File

## Data Availability

The HELIUS data are owned by the Academic Medical Center (AMC) in Amsterdam, the Netherlands. Any researcher can request the data by submitting a proposal to the HELIUS Executive Board as outlined at http://www.heliusstudy.nl/en/researchers/collaboration. Requests for further information and proposals can be submitted to heliuscoordinator@amsterdamumc.nl. The HELIUS Executive Board will check proposals for compatibility with the general objectives, ethical approvals and informed consent forms of the HELIUS study, and potential overlap with ongoing work affiliated with HELIUS. There are no other restrictions to obtaining the data and all data requests will be processed in the same manner.
