# Supplementary material for: Sex differences in CKD risk factors across ethnic groups
Source: Nephrol Dial Transplant. 2024 Feb 8;39(7):1194–7. doi: 10.1093/ndt/gfae038 (PMC11250226; doi:10.1093/ndt/gfae038)
Supplement: gfae038_Supplemental_File [file gfae038_Supplemental_File.docx]

**Suppl. Table 1: Age-adjusted estimated contribution of risk factors to prevalent CKD**

|  | Women | | | | | | | | | | | | | | | Men | | | | | | | | | | | | | | |
| --- | --- | --- | --- | --- | --- | --- | --- | --- | --- | --- | --- | --- | --- | --- | --- | --- | --- | --- | --- | --- | --- | --- | --- | --- | --- | --- | --- | --- | --- | --- |
|  | Overall | | | Dutch | | SA Surinamese | | African Surinamese | | Ghanaian | | Turkish | | Moroccan | | Overall | | | Dutch | | SA Surinamese | | African Surinamese | | Ghanaian | | Turkish | | Moroccan | |
| **CKD Prevalence** | P | PR | PAF | P | PAF | P | PAF | P | PAF | P | PAF | P | PAF | P | PAF | P | PR | PAF | P | PAF | P | PAF | P | PAF | P | PAF | P | PAF | P | PAF |
| Hypertension | 53.6 | 2.7 (2.3-3.2) ^⧫^ | 33.8 | 38.8 | 24.4 | 68.2 | 43.0 | 68.0 | 42.8 | 63.8 | 40.2 | 45.1 | 28.4 | 36.1 | 22.7 | 75.8 | 3.7 (3.0-4.6) | 55.3 | 75.9 | 55.4 | 83.9 | 61.2 | 74.4 | 54.3 | 89.1 | 65.0 | 65.9 | 48.1 | 62.9 | 45.9 |
| Diabetes Mellitus | 21.6 | 2.6 (2.2-3.1) ^⧫^ | 13.4 | 3.7 | 2.3 | 38.5 | 23.8 | 23.8 | 14.7 | 12.1 | 7.5 | 15.5 | 9.6 | 27.2 | 16.8 | 34.3 | 2.9 (2.4-3.5) | 22.5 | 15.2 | 10.0 | 58.1 | 38.2 | 31.1 | 20.4 | 18.2 | 12.0 | 26.8 | 17.6 | 40.3 | 26.5 |
| Smoking* | 16.6 | 0.9 (0.8-1.1) |  | 24.7 |  | 17.1 |  | 26.7 |  | 4.4 |  | 26.6 |  | 2.5 |  | 31.6 | 1.1 (0.9-1.3) |  | 30.4 |  | 42.7 |  | 32.2 |  | 1.8 |  | 37.0 |  | 29.0 |  |
| Obesity | 48.4 | 1.9 (1.7-2.2) | 22.8 | 14.8 | 7.0 | 42.3 | 19.9 | 59.2 | 27.8 | 52.6 | 24.7 | 58.5 | 27.5 | 48.7 | 22.9 | 32.0 | 2.0 (1.7-2.4) | 16.0 | 26.6 | 13.3 | 30.6 | 15.3 | 33.3 | 16.7 | 18.2 | 9.1 | 36.6 | 18.3 | 45.9 | 22.9 |
| **eGFR <60 ml/min/1.73 m^2^ (%)** | | | | | | | | | | | | | | | | | | | | | | | | | | | | | | |
| Hypertension | 76.3 | 2.8 (1.8-4.4) | 49.3 | 55.2 | 35.7 | 85.7 | 55.4 | 92.9 | 60.0 | 93.8 | 60.6 | 60.0 | 38.8 | 53.8 | 34.8 | 80.2 | 2.9 (1.9-4.6) | 52.7 | 77.1 | 50.7 | 79.4 | 52.2 | 77.8 | 51.2 | 93.3 | 61.4 | 81.8 | 53.8 | 77.8 | 51.2 |
| Diabetes Mellitus | 34.4 | 2.4 (1.6-3.4) | 19.8 | 6.9 | 4.0 | 57.1 | 32.9 | 46.4 | 26.7 | 25.0 | 14.4 | 10.0 | 5.8 | 38.5 | 22.2 | 32.8 | 1.9 (1.3-2.7) | 15.3 | 17.1 | 8.0 | 58.8 | 27.3 | 29.6 | 13.8 | 13.3 | 6.2 | 18.2 | 8.5 | 55.6 | 25.9 |
| Smoking* | 9.2 | 0.6 (0.3-1.0) |  | 6.9 |  | 11.4 |  | 10.7 |  | 0.0 |  | 20.0 |  | 7.7 |  | 22.9 | 0.8 (0.5-1.1) |  | 17.1 |  | 35.3 |  | 33.3 |  | 0.0 |  | 9.1 |  | 22.2 |  |
| Obesity | 53.4 | 1.7 (1.2-2.4) | 21.9 | 20.7 | 8.5 | 54.3 | 22.3 | 75.0 | 30.8 | 62.5 | 25.6 | 80.0 | 32.8 | 46.2 | 18.9 | 28.2 | 1.6 (1.1-2.4) | 10.6 | 20.0 | 7.5 | 23.5 | 8.8 | 29.6 | 11.1 | 20.0 | 7.5 | 54.5 | 20.4 | 55.6 | 20.9 |
| **ACR ≥ 3 mg/mmol** | | | | | | | | | | | | | | | | | | | | | | | | | | | | | | |
| Hypertension | 48.8 | 2.9 (2.4-3.4) | 32.0 | 28.8 | 20.0 | 62.6 | 42.4 | 60.0 | 41.9 | 57.9 | 40.0 | 42.6 | 29.1 | 35.5 | 23.0 | 71.8 | 4.4 (3.5-5.6) | 55.5 | 73.1 | 57.7 | 83.2 | 66.2 | 71.1 | 58.7 | 88.9 | 68.4 | 56.0 | 50.3 | 58.3 | 50.0 |
| Diabetes Mellitus | 20.8 | 2.8 (2.3-3.4) | 13.4 | 3.8 | 2.4 | 36.1 | 23.1 | 21.5 | 13.8 | 11.2 | 7.1 | 16.2 | 10.3 | 26.3 | 16.8 | 36.1 | 3.5 (2.9-4.3) | 25.6 | 13.5 | 9.6 | 59.8 | 42.3 | 34.2 | 24.3 | 17.8 | 12.6 | 28.0 | 19.9 | 40.0 | 28.4 |
| Smoking* | 17.8 | 1.0 (0.8-1.2) |  | 34.0 |  | 18.7 |  | 29.5 |  | 4.8 |  | 27.1 |  | 2.6 |  | 32.9 | 1.1 (0.9-1.4) |  | 34.6 |  | 44.9 |  | 30.3 |  | 2.2 |  | 39.2 |  | 28.3 |  |
| Obesity | 48.3 | 2.0 (1.7-2.3) | 24.2 | 11.3 | 5.7 | 40.7 | 20.4 | 57.7 | 28.9 | 51.4 | 25.7 | 57.4 | 28.7 | 48.0 | 24.0 | 33.6 | 2.2 (1.8-2.7 | 18.1 | 30.8 | 16.6 | 33.6 | 18.1 | 34.2 | 18.5 | 17.8 | 9.6 | 34.7 | 18.7 | 45.8 | 24.7 |

**P**: Prevalence of any positive risk factor among participants with prevalent CKD (%). **PR**: Age-adjusted prevalence ratio and 95% confidence interval of CKD between participants with and without risk factor in men and women in the total population. **PAF**: Population attributable fraction, calculated as P((PR-1)/PR) x 100. * PAF for smoking not calculated, PR was smaller than one in women and several ethnic groups. ^⧫^ Significant interaction between Hypertension, Diabetes Mellitus, obesity and sex in the association with CKD prevalence, all p-values < 0.001.

**Suppl. Table 2: Age-adjusted combined estimated contribution of the combined risk factors to prevalent CKD**

|  | Women | | | | | | | | | | | | | | | Men | | | | | | | | | | | | | | |
| --- | --- | --- | --- | --- | --- | --- | --- | --- | --- | --- | --- | --- | --- | --- | --- | --- | --- | --- | --- | --- | --- | --- | --- | --- | --- | --- | --- | --- | --- | --- |
|  | Overall | | | Dutch | | SA Surinamese | | African Surinamese | | Ghanaian | | Turkish | | Moroccan | | Overall | | | Dutch | | SA Surinamese | | African Surinamese | | Ghanaian | | Turkish | | Moroccan | |
|  | P | PRadj | PAF | P | PAF | P | PAF | P | PAF | P | PAF | P | PAF | P | PAF | P | PRadj | PAF | P | PAF | P | PAF | P | PAF | P | PAF | P | PAF | P | PAF |
| Hypertension | 52.1 | 2.9 | 34.1 | 37.1 | 24.5 | 65.0 | 42.9 | 65.2 | 43.0 | 60.8 | 40.1 | 43.2 | 28.5 | 35.9 | 23.7 | 72.8 | 4.2 | 55.5 | 77.6 | 59.0 | 82.1 | 62.4 | 71.4 | 54.3 | 85.7 | 65.1 | 58.2 | 44.2 | 56.5 | 42.9 |
| Diabetes | 21.5 | 2.6 | 7.1 | 4.2 | 1.4 | 38.0 | 12.5 | 23.8 | 7.8 | 11.2 | 3.7 | 15.8 | 5.2 | 27.6 | 9.1 | 34.0 | 2.9 | 22.4 | 13.4 | 8.8 | 59.0 | 38.9 | 31.4 | 20.7 | 17.1 | 11.3 | 27.8 | 18.3 | 40.3 | 26.6 |
| Obesity | 48.5 | 1.9 | 22.8 | 14.1 | 6.6 | 42.1 | 19.8 | 57.9 | 27.2 | 52.0 | 24.4 | 57.6 | 27.1 | 48.1 | 22.6 | 31.9 | 2.0 | 15.9 | 25.4 | 12.7 | 32.5 | 16.3 | 32.4 | 16.2 | 18.6 | 9.3 | 36.7 | 18.4 | 45.9 | 22.9 |
| **Total sum** | **55.4** | | | **27.3** | | **68.9** | | **67.4** | | **57.5** | | **50.9** | | **50.9** | | **79.9** | | | **69.0** | | **97.7** | | **77.7** | | **75.5** | | **70.8** | | **79.0** | |

**P**: Prevalence of any positive risk factor among participants with prevalent CKD (%). **PRadj**: Prevalence ratio and 95% confidence interval of CKD between participants with and without risk factor in men and women in the total population, with adjustment for age and remaining risk factors. **PAF**: Population attributable fraction (%), calculated as P((PR-1)/PRadj) x 100. **Total sum**: Aggregate of the risk factor adjusted PAFs to depict overall contribution of risk factors combined.

***Suppl. Table 3: Age-adjusted estimated contribution of risk factors to prevalent CKD using the CKD-EPI 2021 equation***

|  | Women | | | | | | | | | | | | | | | Men | | | | | | | | | | | | | | |
| --- | --- | --- | --- | --- | --- | --- | --- | --- | --- | --- | --- | --- | --- | --- | --- | --- | --- | --- | --- | --- | --- | --- | --- | --- | --- | --- | --- | --- | --- | --- |
|  | Overall | | | Dutch | | SA Surinamese | | African Surinamese | | Ghanaian | | Turkish | | Moroccan | | Overall | | | Dutch | | SA Surinamese | | African Surinamese | | Ghanaian | | Turkish | | Moroccan | |
|  | P | PR | PAF | P | PAF | P | PAF | P | PAF | P | PAF | P | PAF | P | PAF | P | PR | PAF | P | PAF | P | PAF | P | PAF | P | PAF | P | PAF | P | PAF |
| Hypertension | 52.1 | 2.9 | 34.1 | 37.1 | 24.5 | 65.0 | 42.9 | 65.2 | 43.0 | 60.8 | 40.1 | 43.2 | 28.5 | 35.9 | 23.7 | 72.8 | 4.2 | 55.5 | 77.6 | 59.0 | 82.1 | 62.4 | 71.4 | 54.3 | 85.7 | 65.1 | 58.2 | 44.2 | 56.5 | 42.9 |
| Diabetes | 21.5 | 2.6 | 7.1 | 4.2 | 1.4 | 38.0 | 12.5 | 23.8 | 7.8 | 11.2 | 3.7 | 15.8 | 5.2 | 27.6 | 9.1 | 34.0 | 2.9 | 22.4 | 13.4 | 8.8 | 59.0 | 38.9 | 31.4 | 20.7 | 17.1 | 11.3 | 27.8 | 18.3 | 40.3 | 26.6 |
| Smoking* | 17.0 | 1.0 |  | 28.2 |  | 18.3 |  | 26.4 |  | 4.1 |  | 27.2 |  | 2.6 |  | 31.5 | 1.1 |  | 31.3 |  | 43.6 |  | 35.2 |  | 1.4 |  | 37.2 |  | 29.0 |  |
| Obesity | 48.5 | 1.9 | 22.8 | 14.1 | 6.6 | 42.1 | 19.8 | 57.9 | 27.2 | 52.0 | 24.4 | 57.6 | 27.1 | 48.1 | 22.6 | 31.9 | 2.0 | 15.9 | 25.4 | 12.7 | 32.5 | 16.3 | 32.4 | 16.2 | 18.6 | 9.3 | 36.7 | 18.4 | 45.9 | 22.9 |

**P**: Prevalence of any positive risk factor among participants with prevalent CKD (%). **PR**: Age-adjusted prevalence ratio and 95% confidence interval of CKD between participants with and without risk factor in men and women in the total population. **PAF**: Population attributable fraction (%), calculated as P((PR-1)/PR) x 100.
